# Thyrotoxic and pheochromocytoma multisystem crisis: a case report

**DOI:** 10.1186/s13256-017-1299-y

**Published:** 2017-06-23

**Authors:** Kodai Suzuki, Takahito Miyake, Hideshi Okada, Fuminori Yamaji, Yuichiro Kitagawa, Tetsuya Fukuta, Ryu Yasuda, Yoshihito Tanaka, Haruka Okamoto, Sho Nachi, Tomoaki Doi, Takahiro Yoshida, Keisuke Kumada, Shozo Yoshida, Hiroaki Ushikoshi, Izumi Toyoda, Shinji Ogura

**Affiliations:** Department of Emergency and Disaster Medicine, Gifu University Graduate School of Medicine, 1-1 Yanagido, Gifu, 501-1194 Japan

**Keywords:** Thyrotoxic crisis, Pheochromocytoma multisystem crisis, Emergency endocrine disease, Intensive care

## Abstract

**Background:**

Thyrotoxic crisis and pheochromocytoma multisystem crisis are rare, life-threatening, emergency endocrine diseases with various clinical manifestations. Here we report a case of a patient who simultaneously developed thyrotoxic crisis and pheochromocytoma multisystem crisis and required intensive cardiovascular management.

**Case presentation:**

A 60-year-old Asian man experienced nausea and vomiting, and subsequently developed dyspnea and cold sweats while farming. His serum free thyroxine, free triiodothyronine, and thyrotropin receptor antibody levels were elevated at 2.9 ng/dL, 7.2 pg/dL, and 4.7 IU/L, respectively. Serum thyrotropin levels were suppressed at less than 0.01 μIU/mL. Thyroid echography demonstrated no thyroid swelling (23 × 43 mm).

A whole body computed tomography was performed for systemic evaluation. This revealed exophthalmos and a mass of size 57 × 64 mm in the anterior pararenal space. Based on these findings, we made an initial diagnosis of thyrotoxic crisis secondary to exacerbation of Grave’s hyperthyroidism. Treatment was begun with an iodine agent at a dose of 36 mg/day, thiamazole at a dose of 30 mg/day, and hydrocortisone at a dose of 300 mg daily for 3 consecutive days. To control tachycardia, continuous intravenously administered propranolol and diltiazem infusions were given. At the same time, small doses of doxazosin and carvedilol were used for both alpha and beta adrenergic blockade. On hospital day 5, his blood pressure and serum catecholamine concentrations (adrenalin 42,365 pg/mL, dopamine 6409 pg/mL, noradrenalin 72,212 pg/mL) were still high despite higher beta blocker and calcium channel blocker doses. These findings contributed to the diagnosis of pheochromocytoma multisystem crisis with simultaneous thyrotoxic crisis. We increased the doses of doxazosin and carvedilol, which stabilized his hemodynamic status. On hospital day 16, metaiodobenzylguanidine scintigraphy showed high accumulation in the right adrenal gland tumor. After retroperitoneal laparoscopic adrenalectomy on hospital day 33, his condition stabilized. He was discharged on hospital day 58.

**Conclusions:**

Since he required more intensive cardiovascular management for thyrotoxic crisis, beta blockade was increased under intensive care unit monitoring even though initial alpha blockade is recommended in pheochromocytoma. When these crises occur simultaneously, cardiovascular management can be very challenging.

## Background

Thyrotoxic crisis and pheochromocytoma multisystem crisis are both rare, life-threatening emergency endocrine conditions with various clinical manifestations. In thyrotoxic crisis, excessive thyroid hormone causes multiple organ failure. Although cardiac function initially increases in thyrotoxic crisis, partly due to increased catecholamine sensitivity, the myocardium becomes exhausted and cardiac function eventually decreases [1]. Pheochromocytoma is a neuroendocrine tumor of the adrenal medulla or extra-adrenal chromaffin tissue that failed to involute after birth. These tumors secrete high amounts of catecholamines. Pheochromocytoma multisystem crisis is caused by sudden excessive catecholamine release. Manifestations include organ failure, blood pressure abnormalities, and high fever [2]. Here we report the case of a patient who developed thyrotoxic crisis and pheochromocytoma multisystem crisis simultaneously and required intensive cardiovascular management.

## Case presentation

A 60-year-old Asian man with hypertension was being treated with 2 mg of candesartan daily. Two years ago, a tumor was incidentally discovered in his right adrenal gland on computed tomography (CT), but he did not receive further evaluation. He had no history of diabetes mellitus, hypercholesterolemia, or obvious paroxysmal atrial fibrillation.

While farming, he experienced nausea and vomiting, and subsequently developed dyspnea and cold sweats. He was transported to a local hospital by private vehicle. On physical examination, his body temperature was 36.8 °C, pulse rate was 160 beats per minute, and systolic blood pressure was approximately 80 mmHg. His respiratory rate was 30 breaths per minute with 50% oxygen saturation on room air. He had orbitopathy but no dermopathy or acropathy. Anteroposterior chest radiographs showed pulmonary congestion. His presenting electrocardiogram (ECG) revealed obvious ST segment elevation in leads V1 through V5. A troponin T sensitive rapid assay was positive. Acute myocardial infarction was suspected and diagnostic angiography was performed. However, no significant stenosis and occlusion were detected in any coronary artery. Since he had persistent hypotension and multiple organ failure, he required transfer to an advanced emergency medical service center for intensive care.

**Fig. 1:**
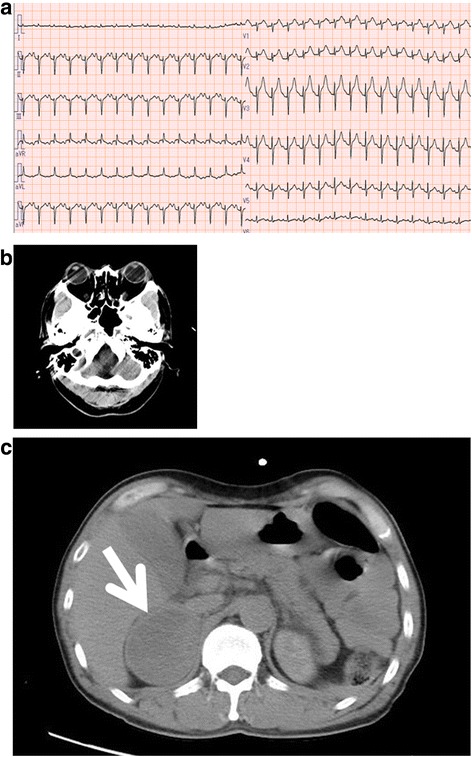
Electrocardiogram and abdominal computed tomography scan findings on admission. **a** Electrocardiogram on arrival at our hospital revealed paroxysmal supraventricular tachycardia. **b** Exophthalmos was observed by computed tomography. **c** Abdominal computed tomography detected a 57 × 64 mm^2^ mass in the anterior pararenal space (*arrow*)

On admission to our institution, his heart rate was 173 beats per minute and blood pressure was 109/85 mmHg with continuous intravenous administration of dopamine (4 μg/kg per minute) and dobutamine (2 μg/kg per minute). An ECG revealed paroxysmal supraventricular tachycardia (Fig. 1a). Echocardiography showed diffuse hypokinesis of his left ventricular wall. Laboratory findings (normal ranges in parentheses) demonstrated a white blood cell count of 22.6 × 10^3^/μL (3.4 to 9.2), serum troponin I level of 43.67 (less than 0.04) ng/mL, creatine kinase level of 5692 (40 to 200) IU/L, aspartate transaminase level of 4236 (7 to 35) IU/L, alanine transaminase level of 3281 (7 to 40) IU/L, lactate dehydrogenase level of 4971 (125 to 225) IU/L, creatinine level of 3.89 (0.60 to 1.20) mg/dL, and blood urea nitrogen level of 49.6 (8 to 20) mg/dL. Serum free thyroxine (T4), free triiodothyronine (T3), and thyrotropin receptor antibody levels were elevated at 2.9 (0.7 to 1.5) ng/dL, 7.2 (1.7 to 3.7) pg/dL, and 4.7 IU/L (less than 15), respectively. His serum thyrotropin levels were suppressed at less than 0.01 (0.35 to 4.94) μIU/mL.

For systemic evaluation, a whole body CT was performed. It revealed exophthalmos (Fig. 1b), pulmonary congestion, and no hemorrhage or infarction in his brain. A mass detected in the anterior pararenal space was consistent with a right adrenal tumor identified 2 years earlier (Fig. 1c).

**Fig. 2:**
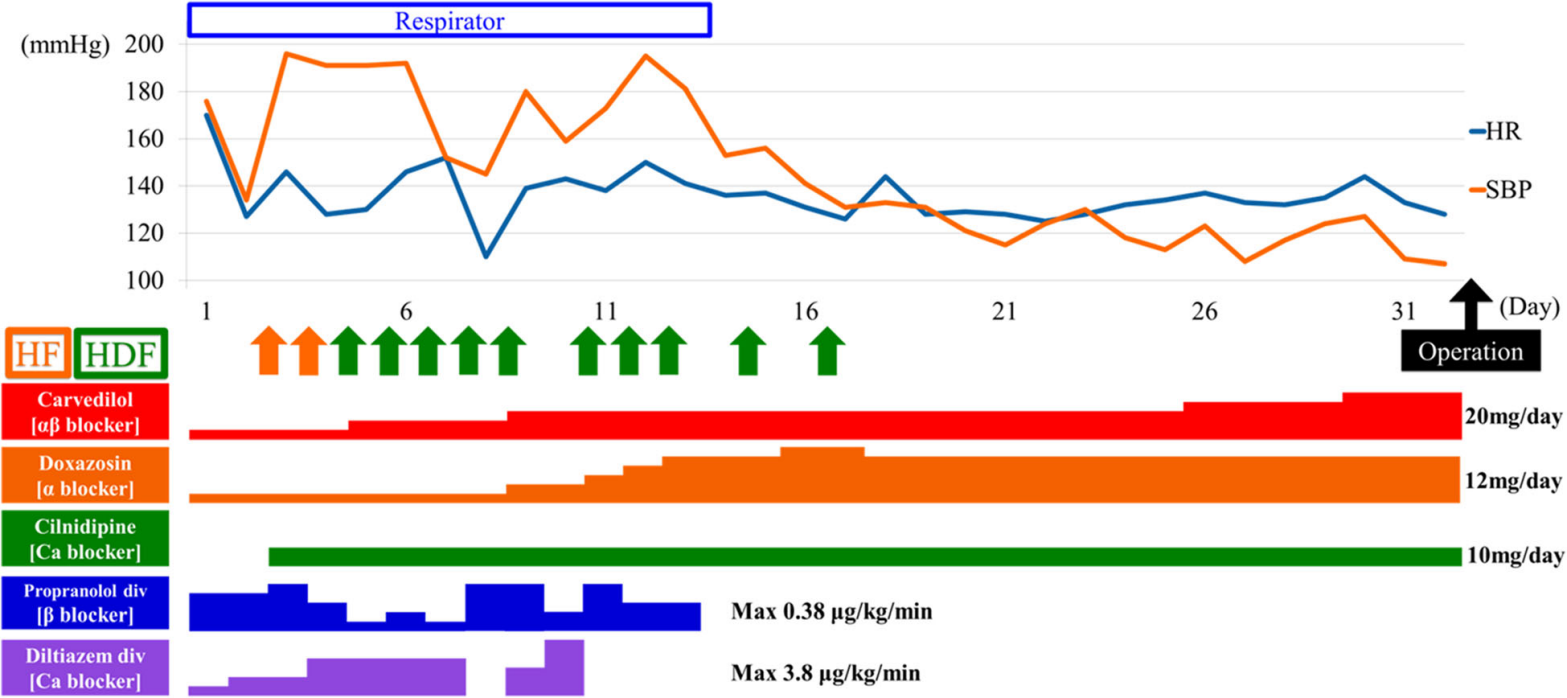
Clinical course. *HDF* hemodiafiltration, *HF* hemofiltration, *HR* heart rate, *SBP* systolic blood pressure

On the basis of these findings, his Burch and Wartofsky score was 95 (Temperature 5, Central nervous effects 20, Hepatogastrointestinal dysfunction 10, Tachycardia 25, Congestive heart failure 15, Arrhythmia 10, Suggestive history 10), and thus we initially diagnosed thyrotoxic crisis secondary to exacerbation of Grave’s hyperthyroidism. Therefore, iodine agent at a dose of 36 mg/day and thiamazole at a dose of 30 mg/day were administered. To reduce peripheral T4 to T3 conversion, hydrocortisone was injected intravenously at a dose of 300 mg daily for 3 consecutive days. To control tachycardia, continuous intravenously administered propranolol and diltiazem infusions were given. At the same time, small doses of doxazosin and carvedilol were used for both alpha and beta adrenergic blockade because we suspected the tumor in the right adrenal gland was a pheochromocytoma (Fig. 2). Next, we considered his systolic blood pressure increase to over 180 mmHg; for blood pressure and heart rate control, orally administered cilnidipine and continuous intravenously administered propranolol and diltiazem were given. Starting on hospital day 3, continuous hemofiltration and hemodialysis were performed because his urine volume decreased and renal failure was aggravated with human atrial natriuretic peptide.

**Fig. 3:**
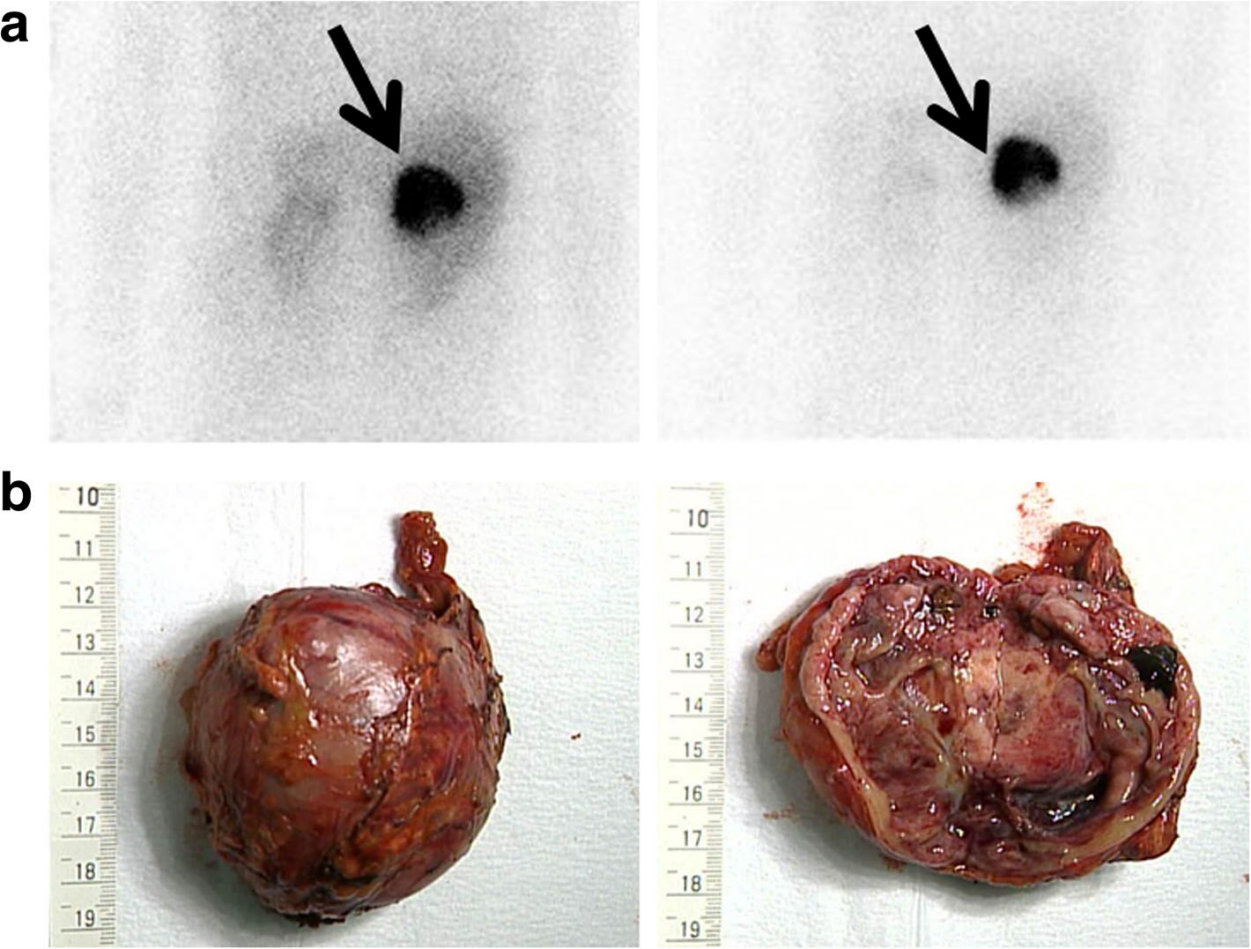
Pheochromocytoma findings. **a** Images taken 6 (*left panel*) and 24 hours (*right panel*) after injection of iodine-131 metaiodobenzylguanidine. Posterior projections of the abdomen revealed foci of moderately increased metaiodobenzylguanidine uptake colocalizing with the adrenal tumor (*arrows*). **b** The pheochromocytoma was resected via retroperitoneal laparoscopic adrenalectomy

On hospital day 5, serum catecholamine concentrations were high despite increased beta blocker and calcium channel blocker doses: adrenalin 42,365 (less than 100) pg/mL, dopamine 6409 (less than 20) pg/mL, and noradrenalin 72,212 (100 to 450) pg/mL. Although the interpretation of these values was complicated by the fact that our patient received noradrenaline and adrenaline for shock at the time of hospital admission, catecholamine assessment helped us diagnose pheochromocytoma multisystem crisis with thyrotoxic crisis. We increased the doses of doxazosin and carvedilol (Fig. 2), which stabilized his hemodynamic status. On hospital day 16, metaiodobenzylguanidine (MIBG) scintigraphy showed high accumulation in the right adrenal gland tumor (Fig. 3a). After retroperitoneal laparoscopic adrenalectomy on hospital day 33 (Fig. 3b), his condition was stabilized. The resected specimen revealed composite pheochromocytoma consisting of pheochromocytoma and ganglioneuroma. He was discharged on hospital day 58.

## Discussion

Grave’s ophthalmopathy can be present with low or normal thyroid hormone levels in the blood, depending on the degree of glandular stimulation induced by autoimmune activity and thyroid gland destruction present at the time of diagnosis. In general, Grave’s disease has existed for 3 to 5 years by the time of diagnosis. Therefore, we hypothesized that this patient already had been affected by Grave’s hyperthyroidism [3]. In addition, a tumor was detected in his right adrenal gland. Since he was asymptomatic, he did not receive any medication as treatment. Subsequent progression suggested that this tumor may be a pheochromocytoma. In the present case, both thyrotoxic crisis and pheochromocytoma multisystem crisis were induced by some factors associated with the patient’s primary illness. Coronary angiography was performed in the local hospital because acute myocardial infarction was suspected before transfer to our center. It has been previously reported that iodinated contrast medium can aggravate hyperthyroidism [4]. In addition, bolus injections of contrast media increase serum adrenaline concentrations in patients with pheochromocytoma [5]. In this patient, there were initially few signs of pheochromocytoma multisystem crisis, and thyrotoxic crisis was already suspected. Contrast media may have aggravated thyrotoxic crisis and induced pheochromocytoma multisystem crisis. Likewise, pheochromocytoma itself can mimic coronary artery syndrome and also lead to renal failure. Since ECG showed obvious ST segment elevation in leads V1 through V5, coronary angiography was indicated. However, we must always keep in mind the risks associated with contrast medium use when medical examinations are performed in patients with undiagnosed hyperthyroidism, pheochromocytoma, and other conditions.

Hyperthyroidism and pheochromocytoma affect the cardiovascular system. Heart failure and tachycardia with atrial fibrillation, which are the typical complications of thyrotoxic crisis, affect circulation kinetics [6]. Pheochromocytoma multisystem crisis also causes numerous cardiovascular abnormalities, including life-threatening ventricular arrhythmias, conduction disturbances, cardiogenic shock, and hypertensive emergency [7].

However, the cardiovascular management strategies for these crises are completely different. Thyrotoxic crisis requires specific thyrostatic treatment and iodine. In addition, beta blockers are used to control tachycardia [8]. Beta blocker therapy prevents catecholamines from binding to beta adrenergic receptors, thereby reducing T4 to T3 peripheral deiodination [8].

On the other hand, establishing the diagnosis of pheochromocytoma multisystem crisis in critically ill patients is challenging. Our patient needed sufficient preoperative treatment to stabilize his hemodynamic status and prevent life-threatening events since the only definitive treatment for pheochromocytoma is surgical resection [9]. Alpha blockade is used to counter the adrenergic effects of catecholamines and enable intravascular volume expansion. Conventionally, beta blockade should never be instituted until alpha blockade has been fully established because unopposed alpha stimulation may lead to severe hypertension in pheochromocytoma [10].

In patients with hypertension secondary to an adrenal tumor, starting antihypertensive agents plus an alpha blocker at the beginning of treatment is desirable. In the present case, alpha and beta blockade were started simultaneously because both thyrotoxic crisis and pheochromocytoma multisystem crisis were suspected. Beta blockade before alpha blockade is definitely contraindicated in the setting of pheochromocytoma, and will itself lead to challenges in the cardiovascular management because of unopposed alpha stimulation. Definitive diagnosis is occasionally delayed as the diagnostic process occurs during emergency intensive care treatment. In fact, this patient’s hemodynamics was unstable and he required urgent care before a definitive diagnosis was made. Since he required more intensive cardiovascular management, beta blockade for thyrotoxic crisis was increased under monitoring in the intensive care unit even though initial alpha blockade is recommended in pheochromocytoma. Thus, this was certainly a case of catecholamine-secreting pheochromocytoma in crisis with further deterioration due to beta blockade before adequate alpha blockade. Before the diagnosis of pheochromocytoma multisystem crisis was confirmed with MIBG scintigraphy [11], alpha blockade was increased and his condition stabilized without major complications.

## Conclusions

This case demonstrates how a double crisis can develop, and offers strategies for cardiovascular management in patients with this condition. Thyrotoxic crisis and pheochromocytoma multisystem crisis are sometimes induced by contrast medium use. When these crises occur simultaneously, cardiovascular management can be very challenging.
